# Functional K_V_10.1 Channels Localize to the Inner Nuclear Membrane

**DOI:** 10.1371/journal.pone.0019257

**Published:** 2011-05-03

**Authors:** Ye Chen, Araceli Sánchez, María E. Rubio, Tobias Kohl, Luis A. Pardo, Walter Stühmer

**Affiliations:** 1 Department of Molecular Biology of Neuronal Signals, Max-Planck-Institute of Experimental Medicine, Göttingen, Germany; 2 Department of Physiology and Neurobiology, University of Connecticut, Storrs, Connecticut, United States of America; University of Muenster, Germany

## Abstract

Ectopically expressed human K_V_10.1 channels are relevant players in tumor biology. However, their function as ion channels at the plasma membrane does not totally explain their crucial role in tumors. Both in native and heterologous systems, it has been observed that a majority of K_V_10.1 channels remain at intracellular locations. In this study we investigated the localization and possible roles of perinuclear K_V_10.1. We show that K_V_10.1 is expressed at the inner nuclear membrane in both human and rat models; it co-purifies with established inner nuclear membrane markers, shows resistance to detergent extraction and restricted mobility, all of them typical features of proteins at the inner nuclear membrane. K_V_10.1 channels at the inner nuclear membrane are not all transported directly from the ER but rather have been exposed to the extracellular milieu. Patch clamp experiments on nuclei devoid of external nuclear membrane reveal the existence of channel activity compatible with K_V_10.1. We hypothesize that K_V_10.1 channels at the nuclear envelope might participate in the homeostasis of nuclear K^+^, or indirectly interact with heterochromatin, both factors known to affect gene expression.

## Introduction

K_V_10.1 (Ether-à-go-go-1, *KCNH1*) is a voltage-gated potassium channel expressed almost exclusively in the mammalian central nervous system, where its physiological roles have not been clearly identified. Intriguingly, over 70% human tumors of various origins show K_V_10.1 expression [1], [2], [3], [4], [5], [6], [7] the intensity of which correlates negatively with survival in some sarcomas [8] and leukemias [9]. It has also been shown that K_V_10.1 not only plays a role in oncogenic transformation and malignancy, but also that the proliferation of cancer cells is dependent on its expression [10]. It nevertheless remains unclear if K^+^ permeation is required for the carcinogenic properties of K_V_10.1. On one hand, a K_V_10.1 open channel blocker [11] and a monoclonal antibody against K_V_10.1 [12] both of which inhibit ion flow, are able to reduce xenograft tumor growth in immunodeficient mice. On the other hand, the Drosophila ortholog eag is also known to induce an increase in cell proliferation independently of ion influx [13], and a non-conducting K_V_10.1 point mutant still promotes tumor progression, although to a reduced extent [11]. Also, not every K^+^ channel shows a similar behavior. For example, K_V_10.5, the closest relative to K_V_10.1, possesses very similar electrophysiological properties, but is not carcinogenic [11].

Interactions of the channels with signaling cascades within the cell could explain these effects without ion permeation. This is in line with the observation that the vast majority of K_V_10.1 protein remains in intracellular pools, including the perinuclear region, in either heterologous systems [14], neurons [15] or tumor cells [16]. It has also been reported that K_V_10.1 plays a role in oxygen homeostasis and angiogenesis independently of K^+^ flow [11], which would provide an advantage to tumor cells but does not explain the observations reported in vitro.

During the eukaryotic interphase, nucleoplasm and cytoplasm constitute two distinct compartments separated by the nuclear envelope, and trafficking between compartments is allowed by the nuclear pore complex (NPC). Small (<40 KDa) soluble proteins can cross the NPC freely, while larger molecules are active and selectively transported [17]. The nuclear envelope is a membranous structure composed of two layers, the inner and the outer nuclear membranes (INM and ONM, respectively), and the space between is termed the perinuclear space. The ONM is an extension of the ER but shows a distinct protein composition, different from that of the rest of the reticulum [18]. Consequently, the perinuclear space is continuous with the lumen of the ER. The protein composition of the INM is radically different from that of the ONM and the ER. Only a handful of transmembrane proteins are known to reach the INM [19].

As a particular type of complex transmembrane protein, ion channels have also been found in the nuclear envelope [20]. Most reports refer to channels in the ONM, while, to our knowledge, only few channels have been reported to date at INM. Marchenko et al. [21] describe the inositol 1,4,5-trisphosphate (InsP_3_) receptor, Longin et al. [22] a zinc and a calcium channel, Rousseau et al. [23] report two chloride conductances and a non-selective cation channel, and Fedorenko et al., describe a large conductance non-selective cation channel in both the ONM and the INM [24]; among all of them, only the InsP_3_ receptor has an identified molecular identity.

The permeability of the NPC to small ions is still under debate. If it would not be permeable to ions, the ionic composition should be different between the nucleoplasm and cytoplasm, and there could be an electrical potential across the nuclear envelope. However, there are few reports on the ion composition of the nucleoplasm [25], [26] and, in most cases, the intranuclear potential is only slightly negative compared to the cytoplasm [20] but is still dependent on the cytoplasmic K^+^ concentration [27]. On the other hand, there are compelling electrophysiological studies where it has been proposed that the NPC can close in a voltage-dependent manner in the absence of cargo, and that ion flow is prevented when the pore is occluded by cargo [28]. This makes it possible for channels at the INM to dampen or amplify cytoplasmic Ca^2+^ transients into the nucleoplasm [29]. From an experimental point of view, the existence of a diffusion barrier also allows measurement of currents from other smaller channels on either the outer or the INM. It should also be noted that the activity of channels at the INM could participate in the regulation of ion exchange between the nucleoplasm and the perinuclear space, which is continuous with the ER lumen but not the cytosol. In any case, it cannot be ruled out that such ion channels may have functions independent of ion permeation.

In the present report, we provide evidence showing that K_V_10.1 is located to the INM and that currents compatible with K_V_10.1 can be detected by patch clamp at the INM. We also show that INM K_V_10.1 might interact with heterochromatin. We discuss these findings in light of the role of K_V_10.1 in cancer, as the INM has been shown to affect gene expression, genome stability and cell senescence, all of which are relevant to cancer [30].

## Results

### K_V_10.1 localizes to the perinuclear region of native and transfected cells

**Figure 1:**
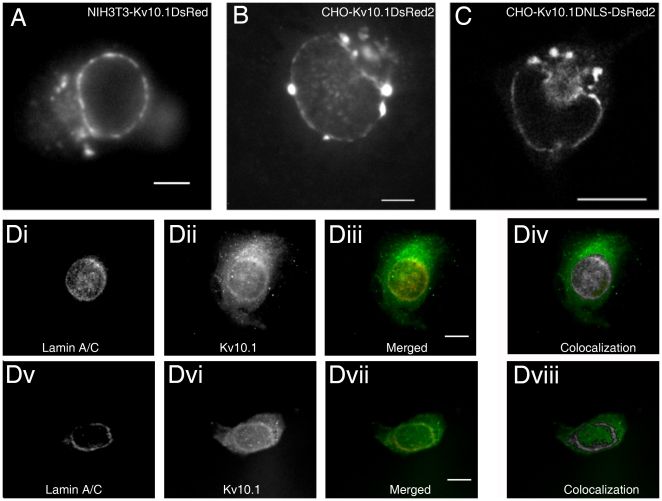
Examples of perinuclear localization of K_V_10.1. A. NIH3T3 cells transfected with K_V_10.1-DsRed (wide field epifluorescence image of living cell, 40x/0.75 Zeiss Achroplan water immersion objective). B. CHO cell transfected with K_V_10.1-DsRed2 (wide field epifluorescence image of living cell, 63x/0.9 Zeiss Achroplan water immersion objective). Scale bars: 5 µm. C. Confocal image showing perinuclear localization of EGFP-K_V_10.1DNLS (devoid of nuclear localization signal) in a living CHO cell (Zeiss LSM510Meta, Plan-Neofluar 40x/1.3 Oil DIC objective; scale bar: 10 µm). D. Two examples of co-localization of K_V_10.1 with lamin A/C. HeLa cells fixed with methanol were stained with goat anti human lamin A/C (Di, Dv; Alexa Fluor 546 donkey anti-goat secondary antibody) and rabbit polyclonal anti-K_V_10.1 (Dii, Dvi; donkey anti-rabbit FITC conjugated secondary antibody) and antibodies. Diii, Dvii merged images. Div, Dviii, Co-localization display; grayscale intensity indicates colocalization (analyzed using FiJi). Wide field epifluorescence image, 63x Zeiss Plan Apochromat 1.4 oil immersion objective. Scale bar: 10 µm. Gaussian blur (d = 2 pixel) was applied to all the images.

There is compelling electrophysiological, microscopical and biochemical evidence that K_V_10.1 is a plasma membrane channel. However, immunostainings of neurons, transfected cells, tumors and tumor cells, as well as in vivo observation of K_V_10.1 labeled with fluorescent proteins (DsRed2, EGFP and mVenus) in different cell types (NIH3T3, CHO, HEK293), revealed perinuclear localization of the protein in 53% of ts20 cells (n = 156) and 11% of CHO cells (n = 203) in the form of a thin line compatible with the nuclear envelope (Fig. 1A, B). Depending on the cell line used, there were roughly 20–40% of cells exhibiting a perinuclear staining pattern possibly indicating an unrevealed role of K_V_10.1 in a particular cellular function.

To confirm this observation, we performed co-localization experiments with lamin A/C as a nuclear envelope marker using double staining with antibodies in HeLa cells expressing endogenous K_V_10.1. A significant level of co-localization was observed (Fig. 1D). This localization is not dependent on Golgi integrity, since it is not abolished by brefeldin A (50 µM) treatment, nor does it entirely co-localize with the ER, as revealed by co-transfection of DsRed-tagged K_V_10.1 and a YFP-ER marker (data not shown). The C-terminus of K_V_10.1 exhibits a bipartite nuclear localization signal. We deleted this sequence to test its influence in the perinuclear localization of K_V_10.1. For this purpose, we used an EGFP-tagged version of K_V_10.1. The deleted mutant still preserves electrophysiological activity when expressed in Xenopus oocytes (data not shown). However, we did not observe abolition of the perinuclear signal (Fig. 1C), and therefore concluded that the identified nuclear localization signal of K_V_10.1 is not required for its targeting to the nuclear envelope.

In heterologous systems and tumor cells, although not in the brain, it could be argued that the expression of the channel in the nuclear envelope is an artifact of massive overexpression. If this were the case, one would expect a positive correlation between overall expression levels and INM expression. However, detailed image analysis revealed that the perinuclear fluorescence intensity of K_V_10.1 did not correlate with cytoplasmic intensity: 91% of ts20 cells showed higher perinuclear than cytoplasmic fluorescence intensity in the immediately neighboring region (n = 90). In 19% of cells, the perinuclear intensity was at least twice as high than the cytoplasmic one (see examples in Figure 1). Inhibition of *de novo* protein synthesis by cycloheximide (10 µg/mL for 1–12 hours) did not abolish the perinuclear localization, also arguing against an overexpression artifact (data not shown). This suggests that, even in overexpression systems, the localization of K_V_10.1 to the nuclear envelope involves a specific regulatory mechanism.

**Figure 2:**
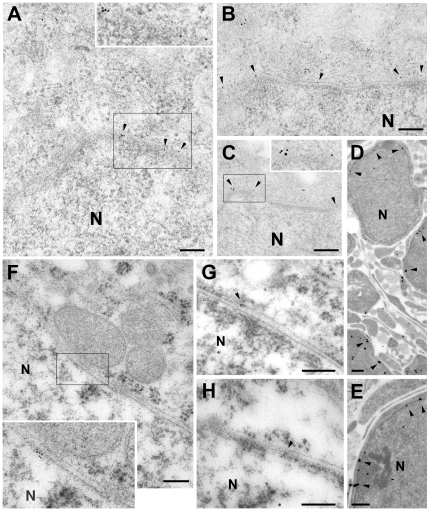
Electron micrographs show postembedding immunogold labeling for K_V_10.1 in MCF-7 cells (A–C), postnatal day 7 cerebellum (D–E) and adult pyramidal cells in CA1 (F–H). Gold particles are observed in the perinuclear inner membrane and/or adjacent to the ONM. N = Nucleus, Scale bars: 0.1 µm.

The proximity of ONM and INM (at the scale of 40 nm [31]) is far beyond the resolution of standard confocal microscopy. We therefore carried out post-embedding immunoelectron microscopy on samples showing endogenous K_V_10.1 expression using mAb62 antibodies. Approximately 90% of gold particles labeling K_V_10.1 channels were detected on the INM of both the human cancer cell line MCF-7 (Fig. 2A–C) and rat cerebellum (Fig. 2D–E) and hippocampus (Fig. 2F–H). The rest of gold particles were localized either in the ONM or in the adjacent cytoplasm.

### The perinuclear localization of K_V_10.1 is compatible with the INM

The ONM is continuous with the ER, where integral membrane proteins are synthesized. These proteins could, in principle, diffuse to the ONM and result in a perinuclear distribution pattern. To distinguish between external and INM localizations, digitonin-permeabilization experiments are often employed. Digitonin preferentially permeabilizes cholesterol-rich membranes (e.g. plasma membrane) and consequently the cholesterol-poor ER and nuclear envelope membranes remain largely intact [32]. In the selectively permeabilized cells, antibodies can only access the cytoplasmic side of ER/ONM proteins but cannot reach the INM proteins, which are shielded by the nuclear envelope. Because the ER lumen is topologically equivalent to the extracellular space, the extracellular domains of K_V_10.1 should face the ER lumen or the perinuclear space, while the C-terminus is expected to face the cytoplasm [33]. Antibodies could therefore not access the INM K_V_10.1, regardless of its orientation, as long as the ONM is intact.

**Figure 3:**
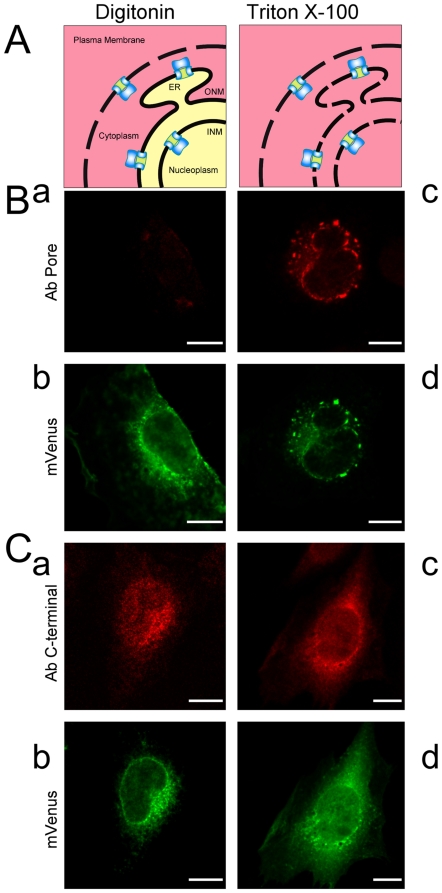
Immunofluorescence microscopy of the intracellular localization of K_V_10.1-mVenus. A. Schematic representation of the topology of K_V_10.1 on different (intra)cellular membranes. Two relevant domains of K_V_10.1 are show: the pore and intracellular C-terminus. Cells in different permeabilized states are shown with red or yellow color representing compartments that are accessible or inaccessible by antibody-containing solution, respectively. In digitonin-permeabilized cells, only the C terminal of ER/ONM K_V_10.1 is accessible while antibodies can reach neither the pore of ER/ONM KV10.1 nor any domain of the INM KV10.1. In Triton-permeabilized cells, all intracellular K_V_10.1 is accessible by antibodies. Transiently transfected CHO cells were permeabilized at 4°C by either digitonin (Ba,b; Ca,b) or Triton X-100 (Bc,d; Cc,d), then fixed and labeled with anti-K_V_10.1 antibody against either the pore (B) or the C-terminal (C). The corresponding fluorescence from the mVenus tagged channel in both treatments is shown in panels b and d. Gaussian blur (d = 2 pixel) was applied to all the images. Scale bar: 10 µm.

CHO cells transiently transfected with K_V_10.1-mVenus were permeabilized either thoroughly with Triton X-100 or selectively with digitonin, and then probed with antibodies recognizing either an extracellular loop (mAb66) or the intracellular C-terminus (mAb33) (Fig. 3). In the Triton X-100 permeabilized cells, signals from the K_V_10.1-mVenus channel perfectly overlapped with the immunofluorescent staining (Fig. 3 Bc,d and Cc,d). In cells permeabilized with digitonin, mAb66 was unable to label the intracellular K_V_10.1 (Fig. 3 Ba,b), suggesting that the integrity of ER/nuclear envelope was preserved, and that the extracellular regions of ER K_V_10.1 face the lumen. In contrast, mAb33 (Fig. 3 Ca,b) mostly stained the intracellular K_V_10.1, but failed to label K_V_10.1 located at the perinuclear rim, indicating that perinuclear K_V_10.1 localized to a subcellular compartment distinct from the ER/ONM but compatible with the INM. Consistent staining patterns were obtained in CHO cells transfected with non-tagged K_V_10.1 (not shown) and are therefore unlikely to be an effect of the mVenus fusion.

**Figure 4:**
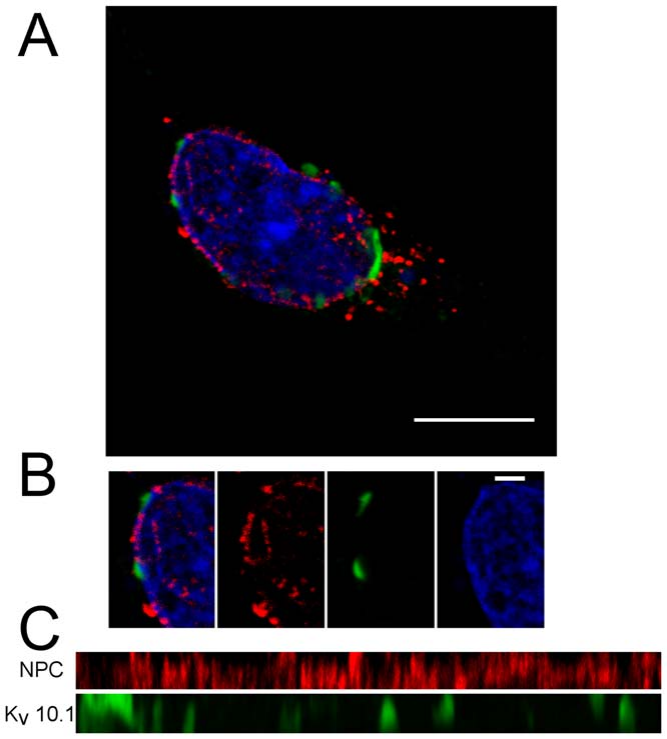
Perinuclear K_V_10.1 is located to NPC-free islands. A. Representative picture of a CHO cell transiently expressing K_V_10.1 and stained with anti-NPC antibodies (red) and anti-K_V_10.1 antibodies (green). In the perinuclear region marked by the boundary of DNA stained by DAPI (blue), K_V_10.1 is excluded from the region containing NPC. Scale bar: 10 µm B. Enlargement of a perinuclear region from the same cell. Scale bar: 2 µm C. The perinuclear region is shown straightened to allow comparison between red and green fluorescence.

During our microscopy experiments, we noticed that perinuclear K_V_10.1 was unevenly distributed, rather than being a smooth line surrounding the whole nucleus. Similar discrete nuclear envelope microdomains, devoid of NPC and therefore termed ‘NPC-free islands’, have already been reported for nurim [34], emerin [35] and the lamin B receptor (LBR) [36], which has been shown to recruit heterochromatin. To test if the patched distribution pattern of K_V_10.1 corresponds to NPC-free islands, we performed double staining of K_V_10.1 and NPC constituents. K_V_10.1 frequently resided at the region of low or no NPC intensity, as indicated by a Manders Coefficient close to 0 (0.27±0.17, n = 15), although NPC is nearly ubiquitous in the nuclear envelope. Perinuclear K_V_10.1 was often found to be close to intense DAPI staining, which typically identifies heterochromatin (Fig. 4), also in live cells expressing KV10.1-mVenus and stained with Hoechst 33342 (not shown).

**Figure 5:**
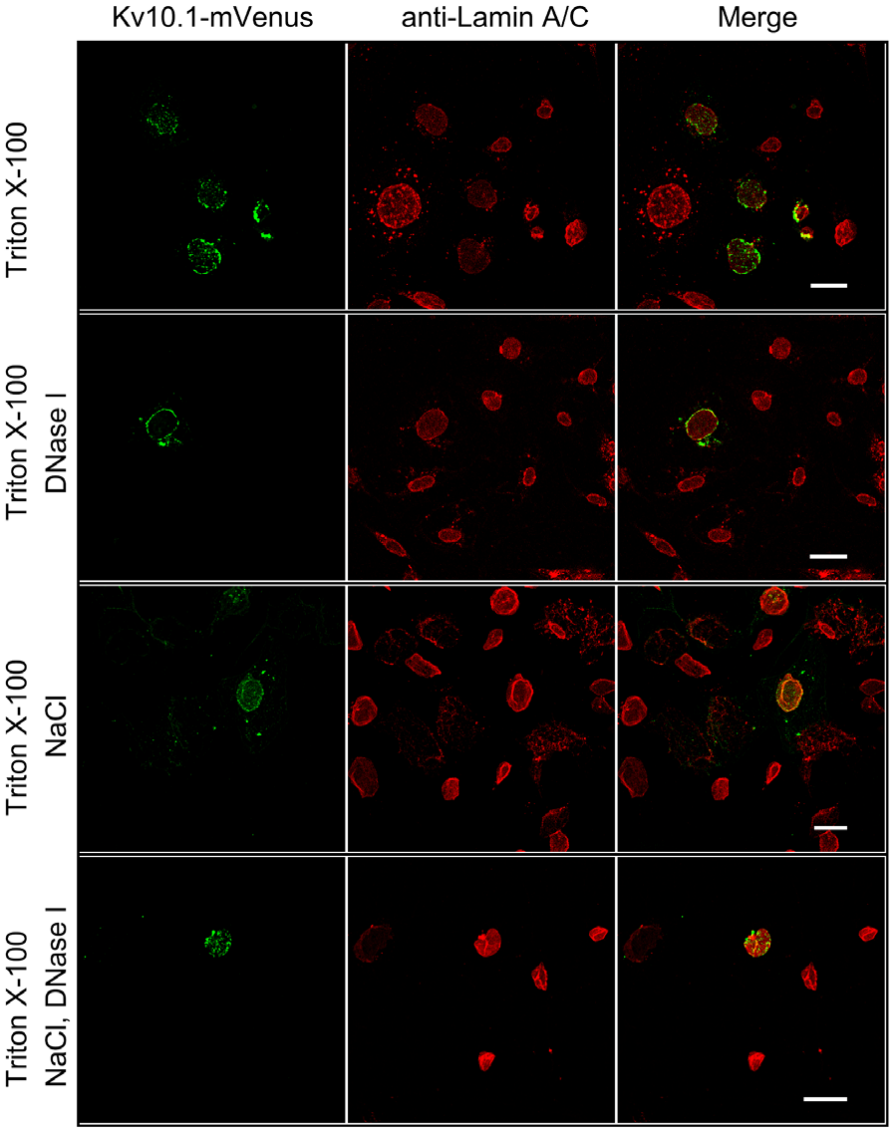
Epitope masking of Lamin A/C in the presence of K_V_10.1. CHO cells transiently expressing K_V_10.1-mVenus (green) were extracted in Triton X-100 (top panel) plus either DNase I (second panel from top), NaCl (third panel) on ice, or DNase I plus NaCl (bottom panel) at 37°C. Cells were then stained with anti-lamin A/C antibodies (red). Note the reduced lamin A/C fluorescence in the upper panel and the rescue in the other three panels. Scale bar, 20 µm.

K_V_10.1 localization to NPC-free islands and proximity to heterochromatin may involve either a direct or an indirect interaction with heterochromatin. INM proteins in direct contact with histones can be detected in the ‘nuclear envelope-peripheral heterochromatin fraction’ [37]. However, this was not the case for K_V_10.1 in the HEK-K_V_10.1 cell line (data not shown) and we therefore tested for indirect interactions. An indication of heterochromatin formation in the case of certain INM proteins is the loss of lamin A/C signals in immunostaining due to epitope masking [38], although it can also be due to phosphorylation of lamin itself [39], [40]. As mentioned before, K_V_10.1 is localized to the same areas as lamin A/C. A closer examination revealed that, within those regions, K_V_10.1 was consistently localized to regions with low lamin A/C fluorescence intensity. In 11/21 cells, K_V_10.1 signal covered 47±5% of the perinuclear region, and there was a masking of local lamin A/C signal but no significant effect on the total intensity (compared to the average value in the same field of view); in 5 cells, K_V_10.1 signal covered 72±8% of the perinuclear region, and there was a global reduction of the lamin A/C fluorescence intensity-typical for “epitope masking”; in the rest of the cells, K_V_10.1 signal occupied 66±10% of the perinuclear region, but there was no obvious effect on lamin A/C signal (Figure 5). This reduction of fluorescence could be rescued partially by extraction with high concentration of salt or incubation with DNase I, and a combination of both treatments resulted in a nearly complete retrieval of lamin A/C fluorescence. This can be interpreted as that the presence of K_V_10.1 correlates with the formation of heterochromatin, either locally or globally, which further masks the epitope of lamin A/C.

### Perinuclear K_V_10.1 is resistant to Triton X-100 extraction and shows restricted lateral mobility

**Figure 6:**
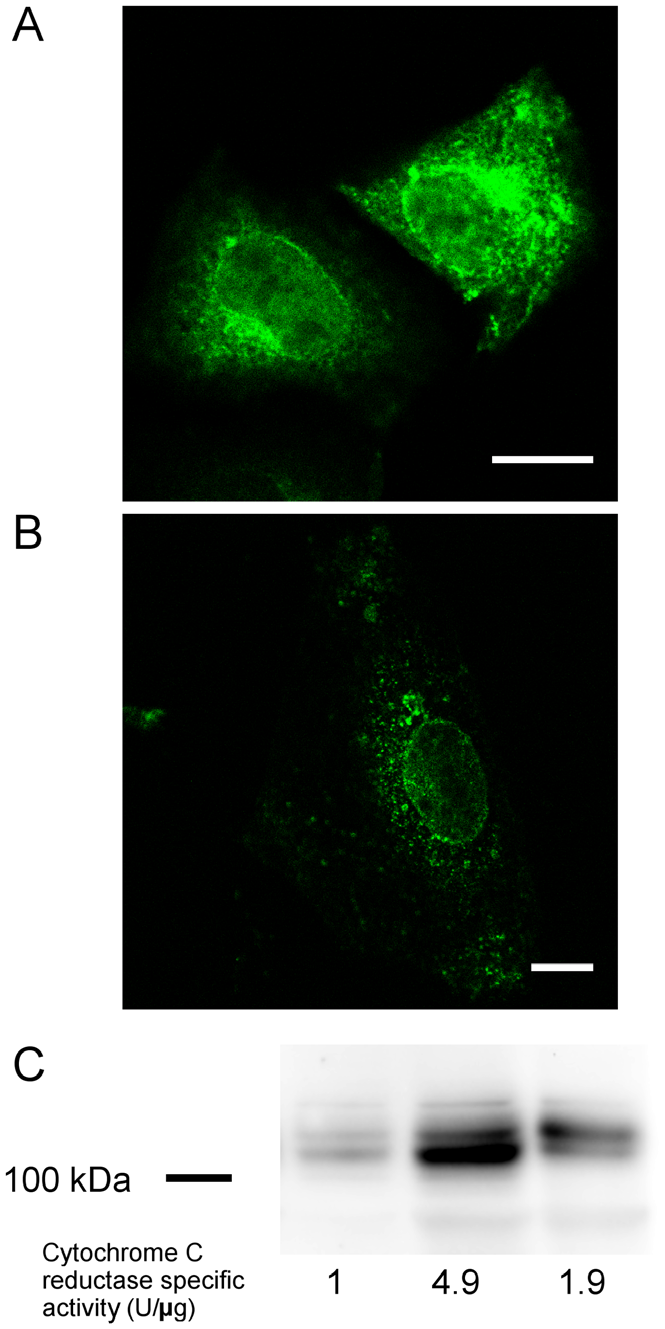
Perinuclear K_V_10.1 is resistant to Triton X-100 extraction. CHO cells transfected with K_V_10.1-mVenus were incubated without (A) or with (B) 3% Triton X-100 for 5 min, fixed and analyzed by confocal microscopy. Triton X-100 largely extracted the cytoplasmic signal, while the perinuclear signal was still detected. Scale bar: 10 µm. C. Western blot analysis using anti-K_V_10.1 antibody on total cell extract, crude nuclear extract and nuclei extracted with 0.5% Triton X-100 from HEK293 cells stably expressing K_V_10.1. Enrichment in nuclear proteins was followed by reduction of NADPH cytochrome c reductase activity.

Higher resistance to non-ionic detergent extraction also characterizes INM proteins. To test this, CHO cells transiently expressing K_V_10.1-mVenus were incubated with extraction buffer containing 3% Triton X-100 before fixation and the fluorescence obtained was examined by confocal microscopy. This extraction removed the fluorescence signals from all the cytoplasm except in the perinuclear region as compared to control cells (Fig. 6A,B). The remaining punctate cytoplasmic structures have been suggested to be aggregates resistant to extraction in similar experiments on other INM proteins [41]. Triton X-100 extraction was also applied to isolated nuclei from HEK-K_V_10.1 cells and evaluated by western blotting. Efficiency of the detergent extraction of the nuclear fraction was tracked by reduction of the ER/ONM marker NADPH cytochrome c reductase. A significant amount of K_V_10.1 remained in the non-extracted (INM) fraction. A fraction of K_V_10.1 was also extracted, possibly representing the ER/ONM (or other contaminating organelles) pool of K_V_10.1 in this overexpression system (Fig. 6B, C).

**Figure 7:**
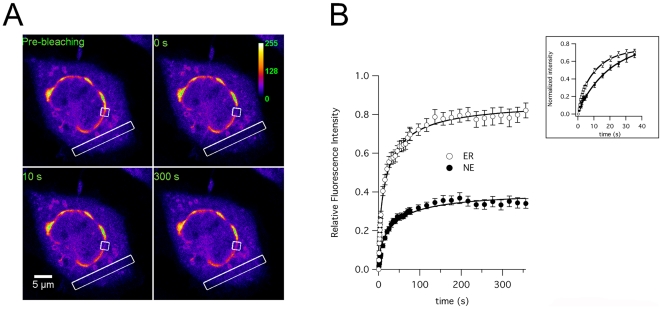
Quantitative FRAP analysis of lateral diffusion of K_V_10.1 in CHO cells transiently expressing K_V_10.1-mVenus. A. Example of the photobleaching process in the perinuclear region and ER (boxes). The recovery is shown at 0, 10 and 300 seconds after bleaching. B, Lower panel shows the average normalized fluorescent intensity during recovery from all cells (±s.e.m.). The first 10 data points were taken every 0.47 seconds, the next 15 every 5 seconds and the last 15 every 20 seconds. The black solid lines are the result of fitting as described in Material and Methods. The initial 40 seconds of recovery are shown normalized in the inset.

INM proteins are typically subjected to various interactions at the lamina layer and their lateral diffusion is therefore restricted as compared to their ER counterparts. To compare the mobility of K_V_10.1 in nuclear envelope and ER, we carried out 1-D FRAP experiments in CHO cells expressing K_V_10.1-mVenus. Two strips (2 µm wide) were bleached at both the nuclear envelope and ER, and the recovery of fluorescence intensity was monitored for 5 minutes. The ER region regained the fluorescence almost completely within 3 minutes, while the nuclear envelope region recovered slowly and only partially (Fig. 7).

Quantitatively, nuclear envelope K_V_10.1 showed a smaller mobile fraction than ER K_V_10.1 (54.3±4.3%, n = 38 compared to 89.9±4.9%, n = 33, p<0.001) and also a reduced diffusion coefficient (4.4±0.8x 10^−3^ µm^2^/s, n = 38 compared to 10.3±2.1x 10^−3^ µm^2^/s, n = 33, p<0.001). These parameters are consistent with those reported for other INM proteins [42], [43], [44], [45] and meet the criteria of an INM-targeted protein. Again, the significant amount of immobile K_V_10.1 and the restricted diffusion of the remaining mobile protein suggest to a tight interaction of K_V_10.1 with its INM environment.

### Subcellular fractionation of K_V_10.1 is compatible with INM localization

**Figure 8:**
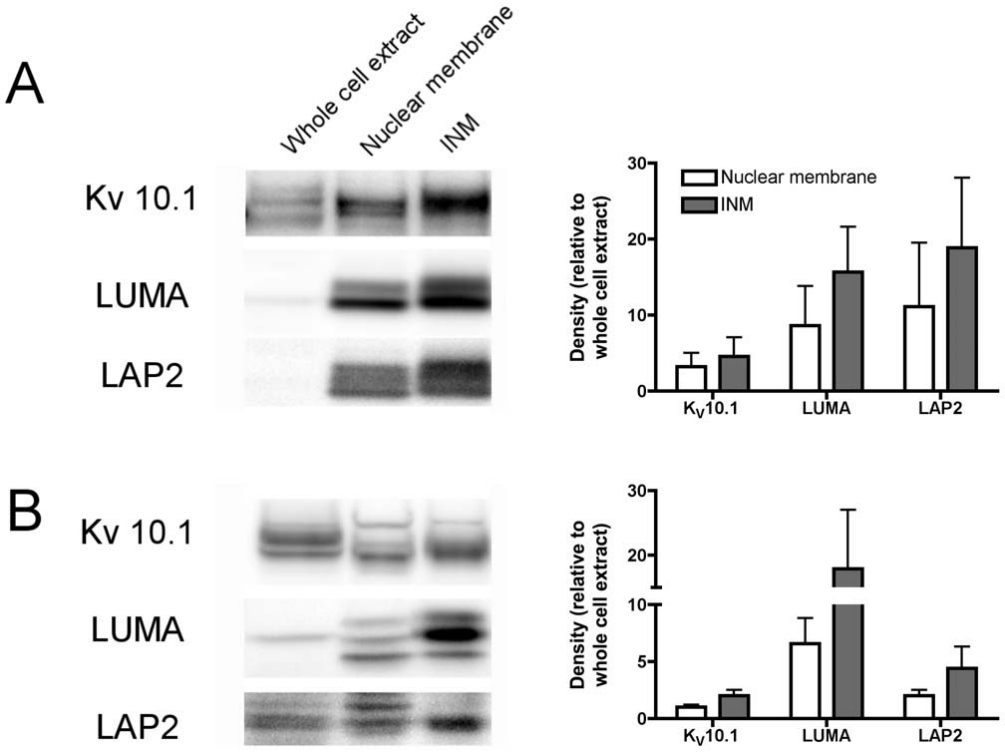
K_V_10.1 proteins can be co-purified from the INM fraction with other INM proteins. The nuclear membrane fraction either from rat brain homogenate (A, n = 3) or HEK293 cells stably expressing K_V_10.1 (B, n = 4) was sedimented by sucrose gradient ultracentrifugation and the ONM was further removed by citraconic anhydride treatment. Proteins from whole cell extract, nuclear fraction (lane 2) and nuclei without the ONM were immunoblotted using anti-K_V_10.1, anti-LUMA and anti-LAP2 antibodies. Quantification corresponds to the relative densitometry measurements for each lane (mean±s.e.m.).

We then set out to confirm the localization of K_V_10.1 to the INM using biochemical tools. We purified nuclei from rat brain as described in the Methods section. After ultracentrifugation through sucrose cushions, nuclei were treated with citraconic anhydride, which is described to specifically remove the ONM [45], [46]. The presence of K_V_10.1 and the well-established INM proteins lamina-associated polypeptide 2 (LAP2) and LUMA was investigated in the different fractions by western blot. K_V_10.1, LAP2 and LUMA were all present predominantly in the nuclear fraction in comparison to the total brain homogenate (Fig. 8A). Removal of the ONM by citraconic anhydride (demonstrated by a>10-fold reduction in cytocrome c reductase activity) resulted in a concurrent enrichment of all three proteins, strongly suggesting that K_V_10.1 is present in the INM in physiological conditions. In transfected HEK293 cells, we also observed that a substantial amount of K_V_10.1 was detected in the nuclear membrane fraction and could be further enriched with LAP2 and LUMA by citraconic anhydride treatment (Fig. 8B).

The relative abundance of K_V_10.1 in nuclear versus extranuclear fractions was much more evident in brain extract than in transfected cells. This is compatible with ‘backed up’ localization previously observed in the overexpression of other INM proteins (LBR [42], MAN1 [47], nurim [41], LUMA [48] and emerin [43]); this phenomenon occurs when an excess of INM proteins saturates the INM retention sites, forcing accumulation in the ER (also observed by immunofluorescence studies in the case of K_V_10.1; data not shown).

**Figure 9:**
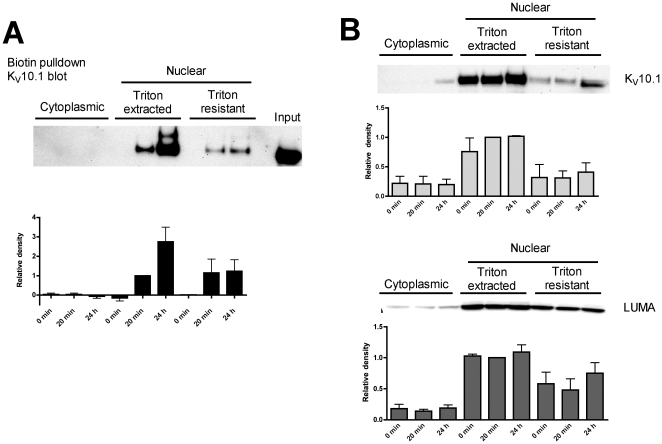
Biotinylated K_V_10.1-AP is detected in the inner nuclear envelope after labeling of the plasma membrane channel. After enzymatic biotinylation, cytoplasmic and nuclear extracts were pulled down (A) with streptavidin-magnetic beads. K_V_10.1-AP was detected in the nuclear fraction and resistant to detergent extraction. As a control, (B) shows the total K_V_10.1-AP and LUMA signals from these extracts before pull-down of biotinylated K_v_10.1-AP. Densitometry for each lane is represented below the corresponding blot (mean±s.e.m, n = 3).

Next, we addressed the questions whether K_V_10.1 at the INM comes directly from the ER, or rather has been properly targeted to the plasma membrane and only then transported to the nuclear compartment. We performed enzymatic surface-biotinylation experiments using a tagged K_V_10.1 channel that carries on an extracellular loop the acceptor peptide (AP) that is recognized and modified by the biotin protein ligase birA [49]. After 20 minutes or 24 hours of biotinylation reaction, the modified proteins were pulled down and analyzed by western blot. Biotinylated K_V_10.1 was preferentially found in the nuclear fractions, and a significant fraction was resistant to Triton X-100 extraction, indicating INM localization (Fig. 9).

Altogether, our results suggest a tight retention of perinuclear K_V_10.1, probably in association with detergent-resistant structures such as lamina or chromatin.

### Single channel activity compatible with K_V_10.1 is detected in the INM

The above-described data do not answer the question whether the channel located at the INM is functionally active. We therefore set up to record single channels from both membranes of isolated nuclei either directly approaching the envelope for ONM measurement or after removal of the outer membrane with citraconic anhydride for INM recordings.

For these experiments, the permeability due to the NPC needs to be suppressed to unmask smaller currents. In the absence of Ca^2+^, which was our initial condition because of the strong inhibition of K_V_10.1 current in the presence of Ca^2+^-calmodulin [50], a large (1 nS) conductance was present in virtually every membrane patch. This current was more evident upon de- or hyper-polarization or mechanical stress. These currents could be inhibited by known nuclear pore current blockers (mAb414, wheat germ agglutinin [51], La^3+^ and Zn^2+^ [52] and are therefore probably due to the NPC. Unfortunately, in our hands neither mAb414 nor wheat germ agglutinin induced complete inhibition. This made us rely on ionic blockers and to remove EGTA from the recording solution, although EGTA was still used throughout the purification process in an attempt to dissociate calmodulin from K_V_10.1. Addition of W-7, a calmodulin antagonist, did not significantly increase the chance of finding an active K_V_10.1 channel (data not shown), suggesting that calmodulin had indeed been effectively removed during EGTA washing. For recording, EGTA was thoroughly washed out and 10 µM La^3+^ was added to the solution. We chose this concentration because it had no evident effect on plasma membrane K_V_10.1, which is also inhibited by extracellular La^3+^ with an apparent IC_50_ of approximately 200 µM.

**Figure 10:**
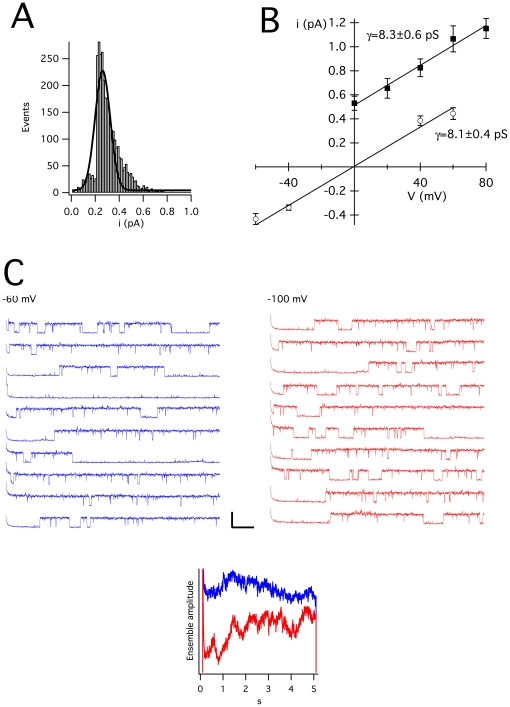
Single channel recording from the INM of HEK-K_V_10.1 cells. A. Amplitude histogram of events recorded at +40 mV in symmetrical potassium (pooled data from 5 independent recordings). B. Single channel amplitudes versus voltage in symmetrical (circles) or asymmetrical potassium (squares). Error bars represent s.e.m. The solid line represents a linear fitting of the data that gives a slope conductance of 8.1 and 8.3 pS. C. Traces recorded at +60 mV from a holding potential of −60 (blue) or −100 mV (red). The latency time before the first opening is increased when the holding potential is more negative, as clearly seen in the ensemble currents depicted in the inset. Scale bars: 2 pA, 500 ms.

**Figure 11:**
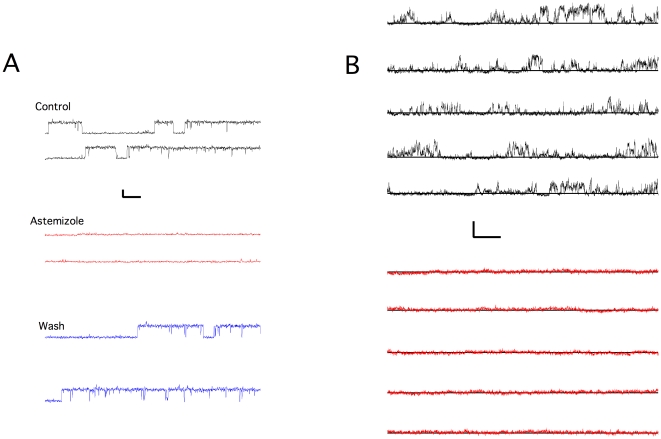
Pharmacological blockade of single channel currents from the INM of HEK-K_V_10.1. A. Block by astemizole. Traces obtained at +60 mV in asymmetrical potassium before (control, black), during (astemizole, red) and after washing (wash, blue) 25 µM astemizole. Scale bars: 2 pA, 500 ms. B. Block by monoclonal antibody mAb56. Control trace, immediately after seal formation at −80 mV in the pipette (black), symmetrical potassium. Red: Recording after 20 minutes incubation at the same voltage Scale bars: 0.5 pA, 500 ms.

In asymmetrical potassium at 0 mV, we detected channel activity with a conductance compatible with K_V_10.1. In symmetrical potassium, large conductance activity was more frequently observed in untreated, ONM preparations. We were unable to record reliable single channel traces from them and they will not be discussed here further. On the other hand, of 64 citraconic anhydride-treated nuclei, 27 records still showed NPC current and were thereby excluded from further analysis, 17 nuclei showed no activity, while the remaining 20 showed comparatively small channel openings. These openings were compatible with K_V_10.1 in terms of conductance (8.1±0.4 pS; Fig. 10A, B) and voltage dependence, with highest open probability at −60 mV and lowest at +60 mV in the pipette, which would represent a membrane potential of opposite sign in an inside-out configuration, that is, if the extracellular side of the channel faces the pipette and the intracellular domains are located to the nucleoplasm. We did not detect any comparable activity in 11 NPC-current-free preparations from non-transfected cells. In asymmetrical K^+^, single channels with voltage dependence similar to the one expected for K_V_10.1, most remarkably the severe dependence of the activation time constant on the prepulse potential described for this channel and commonly used to identify it (see e.g. ref [10]) and a chord conductance of 8.3±0.6 pS was detected (Figure 10 B, C). To characterize the pharmacology of the channel, we used astemizole, a H1 histamine receptor inhibitor often used as blocker of K_V_10.1, because it has not been reported to block conductances other than those of the eag family including K_V_10.1 at a concentration of 2–5 µM. In asymmetrical potassium, we performed continuous recordings at 0 mV to avoid contamination by channels other than those selective for K^+^; under this condition, the activity compatible with K_V_10.1 virtually disappeared in 7/7 patches after application of astemizole. In those cases that allowed washout of the drug (3/7), the activity was recovered. Figure 11 A depicts a similar experiment, but performed at +60 mV to increase current amplitude. In symmetrical K^+^, this treatment reduced the open probability at +60 mV (membrane potential) by 94±10% (n = 5), also indicating a blockade of the channel by astemizole. Since the extracellular side of the channel appeared to be facing the pipette, we used mAb56, a monoclonal antibody reported to bind to the extracellular loops of K_V_10.1 and inhibit current [12]. To do this, we performed layered loading of the pipette by backfilling the antibody-containing solution (45 µg/mL) over antibody-free solution, to allow recording of channel activity prior to the diffusion of the antibody to the pipette tip. The channel activity decreased in all patches tested (n = 6) within 10–20 minutes of the presence of mAb56, a time course compatible with the described action of mAb56 on K_V_10.1 whole-cell current in the plasma membrane (Fig. 11B). Under this experimental setup it is not possible to fully discard a spontaneous rundown of the current, which eventually would happen in any case, but we did not observe rundown in the time range used in these experiments. Due to the selectivity of mAb56, this result strongly supports the molecular identity of the detected channel as K_V_10.1, as well as the proposed orientation in the INM implied by the above experiments, namely that the intracellular C-terminus faces the nucleoplasm.

In addition to the activity attributed to K_V_10.1, it is noteworthy that we also detected other conductances in the range of 20–40 pS, which were unaffected by astemizole or mAb56.

## Discussion

In contrast to traditional ideas, the nuclear envelope is increasingly viewed as a permeability barrier to ions. Although the NPC is generally believed to be responsible for the most of the permeability of the nuclear envelope, there is no correlation between NPC density and the resistivity of the nuclear envelope [20]. In addition, the intranuclear voltage is dependent on cytoplasmic potassium concentration and independent of membrane potential [27]. Therefore, the NPC should be impermeable to small ions in a physiological environment. The ONM, on the other hand, seems to be quite leaky to small molecules, including ions [53]. In this scenario, the structure responsible for the ionic and electrical differences between the nucleoplasm and cytoplasm should be the INM, and ion channels in this membrane would therefore become relevant.

We report here, for the first time, the presence of a functional voltage-gated (the InsP_3_ receptor is a ligand-gated Ca^2+^ channel) ion channel with a defined molecular identity in the INM. This statement is based on both optical and electron microscope data as well as biochemical evidence, both in native and heterologous systems. It not only fulfills all the criteria of a transmembrane protein localized to INM but it is also functional as an ion channel conducting potassium. The evidence in favor of an INM localization include i) inaccessibility to channel-specific antibodies after digitonin permeabilization, ii) resistance to Triton X-100 extraction, iii) limited lateral diffusion, iv) co-segregation with established INM markers and v) electron microscopy. We have used citraconic anhydride to remove the ONM. Other reports have used citric acid or Triton X-100 for the same purpose, but both have the disadvantage that they can also extract INM proteins and make electrophysiological recordings more difficult [54], [55]. Additionally, our electrophysiological experiments indicate the protein orientation; the extracellular loops of the channel face the pipette, which favors localization at the INM. The identity of the channel as K_V_10.1 is supported not only by the fact that it is not found in wild-type nuclei, but also by its voltage dependence-particularly dependence on the prepulse potential-single channel conductance and pharmacology, most importantly by the inhibition measured in the presence of mAb56, which is the most selective blocker available.

We have devoted substantial efforts to make sure that this distribution is not an overexpression artifact. First, INM localization was also observed in native expression systems with immunoelectron microscopy and subcellular fractionation. Second, even in heterologous systems, the expression level in the perinuclear region is not correlated with the level in the cytoplasm, and protein synthesis inhibition does not alter the perinuclear localization, arguing against aberrant relocation of excess K_V_10.1. Third, a significant fraction of the AP tagged protein at the INM has been exposed to the extracellular medium and does not move directly from the ER to the nuclear envelope, arguing against saturation of the transport machinery. We cannot exclude localization also in the ONM, especially in overexpression systems, but the specificity of this localization is more difficult to establish.

Two major types of NLS have been described in the soluble nucleoplasmic proteins SV40 large T antigen and nucleoplasmin. Similar NLS have been predicted in the nucleoplasmic domains of many INM proteins [56] and most of them reside in regions essential for INM localization, as determined by deletion studies (for emerin [43], MAN1 [44] and LEM2 [57], but not for LAP2-β [58]). LBR shows a NLS in its nucleoplasmic domain, but can also be targeted by its transmembrane domain [59]. On the other hand, there is no predicted NLS for nurim, and LUMA has a predicted NLS in its luminal domain [56], and both nurim and LUMA depend on their transmembrane domains for targeting to the INM [34], [48]. SUN-2 bears two NLS at the nucleoplasmic domain, but can be targeted by its luminal domain, which possesses no known NLS [60]. In a similar way, while a conserved bipartite NLS can be identified in the nucleoplasmic domain of K_V_10.1, its deletion however does not significantly alter the INM localization. Since the size of the nucleoplasmic domain of INM proteins able to pass through the NPC during interphase is small (<75 kDa) [56] and the fact that K_V_10.1 is only functional as a tetramer, it is also possible that it is targeted to the INM during reassembly of nuclear envelope in mitosis. However, the relatively fast localization of biotinylated K_V_10.1 to the INM (Fig. 9) would not be explained by such a mechanism.

The function of K_V_10.1 at this location remains only speculative. It could partly underlie the oncogenic properties of the channel. INM proteins play a role in gene expression regulation [61] either by sequestering transcription factors, repressors or other regulators or by direct interaction with chromatin. Within the chromatin in the vicinity of the nuclear envelope, genes encoded in areas close to the NPC are being actively transcribed, while those located away from the NPC (in heterochromatin) are often silent. We did not observe a direct physical interaction of K_V_10.1 with heterochromatin, but the presence of the channel correlated with the absence of NPC and epitope masking of lamin A/C, which is compatible with enrichment in heterochromatin. The physical structure of heterochromatin influences not only transcription efficiency but also splicing [62], [63]. Additionally, K^+^ ions increase the stability of G-quadruplex structures [28], [64] (inter or intramolecular non Watson-Crick pairs in guanine-rich areas), which can act as transcriptional repressor elements (for example of the myc oncogene [65]). There is also abundant evidence of crosstalk between INM proteins and proteins involved in signaling pathways associated with cell cycle or carcinogenesis [66]. Results from our laboratory unequivocally point to a crucial role of K_V_10.1 channels at the plasma membrane, because a functional antibody that blocks the current shows antitumor activity in vivo, and it can exert its action only on exposed channels [12]. The present report indicates a cross talk between the pools of K_V_10.1 at the plasma membrane and at the INM. This is conceptually similar to the role of the C-terminus of a voltage-gated calcium channel, which is cleaved and acts as a transcription factor [67]. There are also descriptions of transmembrane proteins that are translocated from the plasma membrane to the INM [38]. Further experiments to clarify this question are certainly warranted.

## Materials and Methods

### Cell culture and transfection

CHO-K1 (ACC 110), HEK293 (ACC 305), HeLa (ACC 57), MCF-7 (ACC 115), and NIH3T3 (ACC 59) cells (all obtained from DSMZ, Braunschweig, Germany) were grown, respectively, in Ham's F12 Nutrient mix, DMEM:F12, MEM, RPMI1640 and DMEM medium, supplemented with 10% FCS at 37°C in a humidified atmosphere with 5% CO_2_. Cells stably expressing K_V_10.1 (HEK-K_V_10.1 and HEK-K_V_10.1-AP) were cultured in the medium of the respective parental cell lines supplemented with Zeocin (Calya, 3 µg/mL).

EGFP-K_V_10.1 was produced by cloning the open reading frame of K_V_10.1 into pEGFPC2 (Clontech). To do this, an EcoRV site was introduced at the start codon of K_V_10.1. K_V_10.1-DsRed and K_V_10.1-DsRed2 were generated by introducing a SacI site at the termination codon of K_V_10.1 and then cloning the full ORF into pDsRedN1 and pDsRed2N1 (Clontech). The nuclear localization signal was deleted by PCR. The pCDNA3.1 K_V_10.1-mVenus construct was generated by replacing the stop codon of K_V_10.1 by three alanine residues and inserting the coding sequence of mVenus in frame (a mutant Venus-L221K- with less tendency to multimerize). The full coding region was sequenced to ensure that no additional mutation(s) had been introduced.

To mark surface expressed K_V_10.1, the acceptor peptide for biotin ligase (AP) was inserted between the S3 and S4 loops of the channel. This peptide is recognized and biotinylated by the enzyme (BirA), which has only access to exposed parts. The sequence: TGSSGSGSGGLNDIFEAQKIEWHEGGAGGAAGGTG was inserted after residue E317 of K_V_10.1 to produce K_V_10.1-AP.

All tagged-K_V_10.1 exhibit the electrophysiological characteristics of K_V_10.1 in heterologous expression systems (data not shown). For transfection experiments, cells were plated on glass coverslips and transfected at 70–90% confluence using Lipofectamine 2000 (Invitrogen) following the manufacturer's instructions. Immunofluorescence staining and photobleaching experiments were carried out 16–30 hours after transfection.

### Immunofluorescence

Mouse monoclonal and rabbit polyclonal anti-K_V_10.1 antibodies were generated in our laboratory and have been described elsewhere [1]. mAb33 and polyclonal antibodies (2413 and 9391) recognize the C-terminus of K_V_10.1, while mAb62, mAb66 and mAb56 bind to the extracellular S5-S6 linker. For double staining of the NPC and K_V_10.1, a commercial rabbit polyclonal antibody was used (Alomone, Jerusalem, Israel).

Other antibodies used were goat anti human lamin A/C (Santa Cruz Biotechnology, Santa Cruz, CA), mouse anti-NPC monoclonal antibody (mAb414, Abcam, Cambride, UK), rabbit polyclonal anti-LAP2, which recognizes only the transmembrane isoforms in LAP2 family, and anti-LUMA antibodies (kindly provided by Henning Otto, Freie Universität Berlin, Germany).

For double staining of the NPC and K_V_10.1 [35], cells were washed with TBS and fixed with 2% formalin solution (Sigma) for 15 minutes at room temperature. The reaction was quenched with 50 mM glycine for 5 minutes in HMK buffer (20 mM HEPES, 1 mM MgCl_2_, 100 mM KCl, pH 7.5), and the cells rinsed with HMK, permeabilized with 0.5% Triton X-100 (Sigma) in HMK for 5 minutes and incubated in 10% normal goat serum (NGS) in HMK for 30 minutes. The cells were sequentially incubated with rabbit anti-K_V_10.1 (1∶1000, Alomone) in HMK buffer supplemented with 1% NGS, AlexaFluor 488 anti-rabbit antibody (1∶1000, Invitrogen), 1 µg/mL mouse anti-NPC and AlexaFluor 680-labeled anti-mouse antibody (1∶1000 Invitrogen). Between K_V_10.1 and NPC staining, we introduced a blocking step with 10% NGS in HMK for 30 minutes. The coverslips were mounted using DAPI-containing ProLong Gold Antifade reagent (Invitrogen).

Lamin A/C staining and unmasking was performed as described in (Isokane, Hieda et al. 2008) with modifications. Briefly, CHO cells transiently expressing K_V_10.1-mVenus were permeabilized for 10 minutes on ice in 20 mM Tris-HCl, 3 mM MgCl_2_, 0.5 mM CuCl_2_, pH 7.5 with 0.4% Triton X-100. Then the cells were extracted either on ice for 5 minutes with 0.5 M NaCl or at 37°C for 20 minutes with DNase I (Qiagen, 80 U/mL) or DNase I plus 0.5 M NaCl, washed, fixed and then blocked in TBS plus 3% BSA for 1 hour, stained for 3 hours at 37°C against lamin A/C at 1∶100 dilution (Santa Cruz) and probed with anti-goat AlexaFluor secondary antibodies (1∶2000, 1 hour).

### Detergent extraction and permeabilization experiments

For Triton X-100 extraction experiments, CHO cells transfected with K_V_10.1-mVenus were incubated on ice for 5 minutes with or without 3% Triton X-100 in extraction buffer [41] (10 mM HEPES, 80 mM KCl, 16 mM NaCl, 1.5 mM MgCl_2_, 1 mM DTT, 30% glycerol, and protease inhibitor mixture -Complete, Roche Applied Science, pH 7.9). The cells were then fixed in 4% p-formaldehyde at room temperature for 15 minutes and incubated with 50 mM NH_4_Cl in PBS for 10 minutes and mounted.

Digitonin permeabilization experiments were performed as described in [59], with modifications. CHO cells transfected with K_V_10.1-mVenus were fixed with 4% paraformaldehyde for 10 minutes at room temperature and incubated for 10 minutes in 50 mM NH_4_Cl in PBS. After that, the cells were incubated for 5 minutes at 4°C in 40 µg/mL digitonin (diluted from a 20 mg/mL DMSO stock), or 0.5% Triton X-100 in PBS. After a blocking step with 10% BSA in PBS for 30 minutes, the cells were incubated with the desired primary antibodies (either mAb66 or mAb33, 0.5 µg/mL) for 1 h in TBS and anti-mouse AlexaFluor 546 secondary antibodies (1∶1000, 30 minutes).

Fluorescence signals were collected with the equipment indicated in each Figure. Wide field epifluorescence images were obtained in a Zeiss Axioskop 2 microscope equipped with a SPOT 1.3 camera and using the SPOT software. Confocal microscopy was performed in a laser scanning confocal microscope (TCS-SP2; Leica) using an oil immersion objective (HCX PL Apo, 63×/NA = 1.4) or a Zeiss LSM 510 Meta device using a Plan-Neofluar 40x/1.3 Oil DIC objective. ImageJ [68] and Adobe Photoshop were used for offline image processing. No non-linear image modifications were performed. The JACoP plug-in [69] was used to quantify the co-localization of K_V_10.1 and NPC, and the “Straighten Curved Objects” plugin [70] for the straightened view shown in Figure 4C.

### Nuclear isolation, surface labeling and western blot

Whole cell lysates were obtained by homogenization in denaturing lysis buffer (50 mM NaHCO_3_, 15 mM Na_2_CO_3_, 2% SDS). For Triton X-100 extraction experiments, nuclear preparations were obtained using the NucBuster Nuclear Protein Extraction Kit (Novagen, Darmstadt, Germany) according to the manufacturer's manual except for the addition of an extraction step of the nuclear pellet in extraction buffer +0.5% Triton X-100. Cytochrome c assay was performed as described in [71].

For nuclear protein preparation, the nuclei were prepared by homogenizing HEK-K_V_10.1 cells in hypotonic solution or minced rat brain in nuclear isolation medium (NIM, 0.25 M sucrose, 25 mM KCl, 5 mM MgCl_2_, 10 mM Tris/HCl, pH 7.4). The crude nuclear extracts were then washed twice in NIM. The nuclei were resuspended in one volume of NIM and then mixed with two volumes of a sucrose density barrier (SDB: 2.3 M sucrose, 25 mM KCl, 5 mM Tris/HCl, pH 7.4). The whole mixtures were laid on top of SDB and centrifuged at 100,000 xg for one hour. The pellets containing nuclei were subjected to citraconic anhydride to remove the ONM as described by [45]. Then the nuclear proteins were prepared by the low-ionic-strength method [71].

For western blots, proteins were separated in a 3–8% gradient polyacrylamide gel electrophoresis (Invitrogen), transferred to nitrocellulose membranes and probed with K_V_10.1 antibodies (9391). The same membranes were subsequently stripped (Restore Western Blot Stripping Buffer, Thermo Scientific) and re-probed with anti-LAP2 (1∶2000) and anti-LUMA (1∶2000).

To label surface-expressed K_V_10.1-AP by enzymatic biotinylation, stably transfected HEK293 cells were incubated for 20 minutes at 37°C in 10 µM biotin and 120 nM BirA (Avidity, USA) in PBS (containing 4 mM MgCl_2_ and 1 mM ATP). For 24 hours continuous labeling, after the described incubation, culture medium supplemented with 1 mM ATP, 10 µM biotin, 30 mM di-sodium phosphocreatine and 30 U/mL creatine phosphokinase was added to the reaction.

Biotin-labeled proteins were pulled-down in 2 mL NPE lysis buffer (150 mM NaCl, 5 mM EDTA, 50 mM Tris-HCl, 5 mM KCl, 1% NP-40, Complete Protease-inhibitors (Roche), pH 7.5) with 30 µL Dynabeads MyOne Streptavidin T1 (Invitrogen) for one hour at 4°C. The beads were then washed three times in NPE, once in TBS and the proteins were recovered in NuPAGE loading buffer.

### Electrophysiological Recordings

Nuclei from HEK-K_V_10.1 cells were prepared essentially as those for biochemical analysis with the addition of 10 mM EGTA to all solutions. After sedimentation through a 2.3 M sucrose cushion, nuclei were resuspended in NIM for ONM measurements. For INM measurements, nuclei were further treated with citraconic anhydride in modification buffer (200 mM HEPES/NaOH pH 8.5, 1 mM MgCl_2_, 250 mM sucrose) and washed twice in NIM solution. The nuclei were stored at 4°C in NIM and measured within 36 hours of preparation.

Single channel recordings were performed using bath solution 150 mM KCl, 5 mM MgCl_2_, 10 µM LaCl_3_, 10 mM HEPES/KOH pH 7.4. The pipette solution for symmetrical K+ recordings was identical to the bath solution except that 200 µM CaCl_2_ was added to improve seal formation. For recording in asymmetrical K+, the pipette contained 160 mM NaCl, 2.5 mM KCl, 2 mM CaCl_2_, 1 mM MgCl_2_, 10 µM LaCl_3_, 8 mM glucose, 10 mM HEPES/NaOH, pH 7.4. The osmolarity of all solutions was 300–330 mmol/kg. Nuclei in suspension were allowed to attach to the plastic bottom of the experimental chamber or to a poly-L-Lysine- coated coverslip for 5 minutes and were then gravity perfused extensively with bath solution to remove trace amounts of EGTA. Ellipsoid nuclei without attached membrane debris were selected visually for measurement. Currents were recorded using an EPC9 patch-clamp amplifier (HEKA Elektronik, Lambrecht, Germany) in the “nucleus-attached” configuration. This is equivalent to the standard cell-attached configuration for the INM, but outside-out for the ONM. Pulse generation and data acquisition were controlled with Pulse software (HEKA Elektronik). Data were filtered at 1 kHz and digitized at 5 kHz. Patch pipettes were pulled from WPI.PG10165-4 glass (World precision Instruments) or Hilgenberg thick-wall (0.5 mm) borosilicate with a resistance of 7–12 MΩ. Offline data analysis was performed using TAC (Bruxton Corp. Seattle, US.). Astemizole (Sigma) was prepared in 10 mM stock solution in DMSO and used at 1∶5000 dilution shielded from light. Solution exchanges were completed in 5 minutes. For antibody blockage, the tip of pipette was loaded with normal pipette solution and the pipette was then backfilled with 300 nM mAb56. All electrophysiological experiments were performed at room temperature (20–22°C).

### Fluorescence recovery after photobleaching (FRAP)

CHO cells plated on 40 mm coverslips were transfected as described above. Immediately before FRAP experiments, cells were incubated with Hoechst 33342 (50 ng/mL, 5 minutes at room temperature) to stain the nuclei and washed twice with TBS. FRAP was performed using a 40x, HCX PL Fluotar, 1.25 NA oil immersion objective on a Leica TCS SP II confocal microscope installed in a temperature controlled chamber. Parameters were identical in all experiments; cells were kept at 37°C in a Focht Live-Cell Chamber System (Bioptechs Inc, Butler, US.) in extracellular solution (160 mM NaCl, 2.5 mM KCl, 1 mM MgCl_2_, 2 mM CaCl_2_, 8 mM Glucose, 10 mM HEPES/NaOH pH 7.4.). At a spatial sampling frequency of 40 nm, two 2 µm strips either on the perinuclear region or through the cytoplasm were chosen as regions of interest (ROI). The cells were scanned at 1400 Hz with Ar 514 laser at transmission of 7% every 0.47 seconds for 5 times before bleaching as the reference images, in 12-bit, 512×512 pixels format and then ROI were bleached 20 times at 100% transmission. The first 10, 15 and the last 15 post-bleached images were taken every 0.47, 5 and 20 seconds correspondingly. The emission light was collected in the range of 527 nm to 608 nm.

All the images were background-subtracted and then all the post-bleach images were corrected for photobleaching on the principle that the fluorescence intensity of whole post-bleached images should stay the same as the intensity outside the ROI in the reference images. Then the time-lapse images were corrected for lateral drift using StackReg [72].

The fluorescence intensities of the ROIs were normalized to a function of time [73].
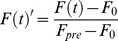
(1)


F (t) is the fluorescence intensity of the ROI at time t. F_0_ is the ROI intensity of the first post-bleach image and F_pre_ is the average ROI intensity of the first 5 reference images. F (t) ' is the ROI fractional fluorescence intensity at time t. The diffusion coefficient D was calculated by fitting the data with [42]:
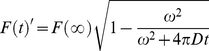
(2) where D is the one-dimensional diffusion constant; ω is the width of the strip or the square, (2 µm in our case). t_0_ was taken as the midpoint of the last bleach. F(∞) is the value of asymptote when t approaches infinity. All the data fitting was processed by Igor Pro 6.0.2 (WaveMetrics, Inc. Lake Oswego, OR).

Based on the fitting curves, the mobile fractions (M_f_) were calculated as:
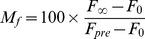
(3) where F_0_ is the fractional intensity at t_0_.

### Freeze-substitution and postembedding immunogold labeling

MCF7 cells (DSMZ, see above) and hippocampal and cerebellar slices from 3 Sprague-Dawley rats (Harlan Research Laboratories) were used for freeze substitution. Cell cultures were immersion fixed with 4% paraformaldehyde and 0.5% glutaraldehyde in 0.1 M phosphate buffer, pH 7.15, for 1.5 hours. Rats for the hippocampal and cerebellar analysis were transcardially perfused and post-fixed with the same fixative. After fixation, a freeze substitution protocol similar to that described previously was used [74], [75]. Hippocampal and cerebellar regions were carefully dissected and processed for freeze-substitution and low-temperature embedding. For post-embedding immunocytochemistry, ultrathin sections (80 nm in thickness) on nickel grids were incubated in sodium borohydride and glycine in Tris-buffered saline solution with Triton X-100. After being pre-blocked with serum, the sections were incubated with affinity-purified primary antibody mAb62 (1 µg/mL; 1∶200 dilution). Primary antibody was detected with secondary antibodies conjugated to 5 nm gold particles (1∶20; Amersham, Arlington Heights, USA). In some ultrathin sections from cerebellum, the gold conjugated secondary antibody was silver intensified using the Aurion silver enhancement kit (Aurion, Wageningen, The Netherlands).The specificity of the antibody has been established previously [1]. Controls included omitting mAb62 and pre-absorption of mAb62 with the corresponding blocking protein (10 µg/mL final concentration). Ultrathin sections were counterstained with uranyl acetate and lead citrate and studied with a transmission electron microscope. Electron micrographs were taken at 30,000x magnification and scanned at a resolution of 3600 dpi using a Linotype-Hell scanner (Heidelberg, Germany). Image processing was performed with Adobe Photoshop using only the brightness and contrast commands to enhance gold particles.

### Ethics

None of the experiments reported here required human material, and therefore ethical approval was not necessary. The experimental procedures on rats were approved and supervised by the University of Connecticut Institutional Animal Care and Use Committee (IACUC-A08.037) in accordance with NIH guidelines.
